# Deconvolution of hemodynamic responses along the cortical surface using personalized functional near infrared spectroscopy

**DOI:** 10.1038/s41598-021-85386-0

**Published:** 2021-03-16

**Authors:** A Machado, Z Cai, T Vincent, G Pellegrino, J-M Lina, E Kobayashi, C Grova

**Affiliations:** 1 Multimodal Functional Imaging Laboratory, Biomedical Engineering Department, McGill University, 3801 Rue University 751, Montreal, QC H3A2B4 Canada; 2 Department of Physics and PERFORM center, Concordia University, Montreal, Canada; 3 Department of Neurology and Neurosurgery, Montreal Neurological Institute, McGill University, Montreal, Canada; 4 École de technologie supérieure de l’Université du Québec, Montreal, Canada; 5 Centre de Recherches en Mathématiques, Montreal, QC Canada; 6 Centre d’Etudes Avancées en Médecine Du Sommeil, Centre de Recherche de l’Hopital Sacré-Coeur De Montréal, Montreal, QC Canada

**Keywords:** Neuro-vascular interactions, Imaging and sensing

## Abstract

In functional near infrared spectroscopy (fNIRS), deconvolution analysis of oxy and deoxy-hemoglobin concentration changes allows estimating specific hemodynamic response functions (HRF) elicited by neuronal activity, taking advantage of the fNIRS excellent temporal resolution. Diffuse optical tomography (DOT) is also becoming the new standard reconstruction procedure as it is more accurate than the modified Beer Lambert law approach at the sensor level. The objective of this study was to assess the relevance of HRF deconvolution after DOT constrained along the cortical surface. We used local personalized fNIRS montages which consists in optimizing the position of fNIRS optodes to ensure maximal sensitivity to subject specific target brain regions. We carefully evaluated the accuracy of deconvolution when applied after DOT, using realistic simulations involving several HRF models at different signal to noise ratio (SNR) levels and on real data related to motor and visual tasks in healthy subjects and from spontaneous pathological activity in one patient with epilepsy. We demonstrated that DOT followed by deconvolution was able to accurately recover a large variability of HRFs over a large range of SNRs. We found good performances of deconvolution analysis for SNR levels usually encountered in our applications and we were able to reconstruct accurately the temporal dynamics of HRFs in real conditions.

## Introduction

Continuous wave functional Near Infrared Spectroscopy (fNIRS) is a non-invasive technique that allows measuring oxygenated (HbO) and deoxygenated (HbR) hemoglobin concentration changes ($\Delta [HbO]$ and $\Delta [HbR]$) within the brain^1^. In fNIRS, near-infrared light sources and detectors (i.e., an optode montage) are placed over the scalp. In the near infrared spectrum HbO and HbR are the two main optical absorbers in cerebral tissue. Assuming homogeneous hemoglobin concentration changes inside a portion of the illuminated volume, the modified Beer Lambert Law is usually considered to convert changes in Optical Density ($\Delta OD$) measured at different wavelengths (690 and 830 nm in this study) to $\Delta [HbO]$ and $\Delta [HbR]$. Topographic maps of $\Delta [HbO]$ and $\Delta [HbR]$ along the scalp surface are then obtained after spatial interpolation of the measurement points of each source-detector pair^2^.

However, this traditional topographic approach is lacking spatial resolution and suffers from imprecise optical pathlength information. On the other hand, Diffuse Optical Tomography (DOT)^3–8^ may be used to convert the variations of Optical Densities ($\Delta OD$) measured on the scalp into hemoglobin concentration changes directly within brain volume or along the cortical surface. DOT relies on the calculation of a realistic forward model that describes the light propagation in the underlying tissues and an accurate regularization strategy to solve the inverse problem^9,10^. DOT allows improving not only the spatial resolution but also the quantification properties of $\Delta [HbO]$ and $\Delta [HbR]$ when compared to the classical modified Beer Lambert Law approach^11,12^. As DOT requires many overlapping measurements to achieve accurate reconstructions, most DOT studies used regular and dense arrays of optodes over extended areas^13,14^, which might be cumbersome in realistic lifestyle conditions or clinical applications. We and others recently demonstrated that using few sources and detectors positioned adequately with an “optimal montage methodology”, maximizing a priori fNIRS sensitivity to targeted brain regions, allows achieving accurate local reconstructions along the cortical surface^15–18^.

Despite its limited spatial resolution and penetration depth, fNIRS has emerged as a complement to functional Magnetic Resonance Imaging (fMRI) due to its portability and possibility to perform long-lasting scans at bedside^8,16,19^. Alike fMRI, we generally assume that there is a linear and time invariant relationship between events of a specific task and the impulse response of the neurovascular system called the Hemodynamic Response Function (HRF). Therefore, $\Delta [HbO]$ or $\Delta [HbR]$ at specific measures can be modeled as a convolution of the experimental paradigm by an HRF. Assuming that the noise has a known temporal covariance structure, parametric generalized linear models are commonly used to estimate the evoked HRFs^20^. In common practice, it is usually accepted to adopt an a priori fixed shape for the HRF^21–28^. For instance, the canonical BOLD-HRF^29,30^ is a standard choice, leaving only magnitude coefficients as free parameters to estimate. This parametrization has the advantage to offer optimal statistical power and interpretability if the HRF shape is specified correctly. However, according to both fMRI and fNIRS literature, there are temporal differences between $\Delta [HbO]$ and $\Delta [HbR]$^31^ and there is evidence that the shape of the HRF varies across subjects, brain regions and tasks^32–37^. Additionally, the HRF may also be altered in some pathological conditions^16,38,39^. For these reasons, fixed HRFs might not be an accurate a priori to model the dynamics of $\Delta [HbO]$ and $\Delta [HbR]$ in fNIRS.

Different approaches have been proposed to attempt estimating the HRF shape through a set of basis functions. Employing the first and second derivatives of the canonical HRF or a combination of gamma functions are the most common ways to capture the variability in the hemodynamic responses^26–28,40,41^. One of the most flexible model, a finite impulse response basis set^42^, contains one free parameter for every time-point of the HRF. It allows to model the whole range of shapes and delays for either $\Delta [HbO]$ or $\Delta [HbR]$ exploiting the richness of the high temporal resolution offered by fNIRS. However, deconvolution is challenging as the high number of parameters may over-fit the noise in the data and may lead HRF shapes that are unrealistic. Several studies have demonstrated that deconvolution analysis is feasible in the sensor space^41,43–45^, but sensitive to physiological confounding factors and noise level^40^. In the context of DOT reconstruction, which is a complex process also dependent on noise level^46^, it is still unclear whether deconvolution can be applied in a robust manner after DOT reconstruction.

The objective of this study was to evaluate the robustness of the deconvolution analysis when applied to DOT reconstructions on personalized fNIRS montages spatially constrained towards a target brain region^15,18^. HRF estimation was obtained using a Maximum Likelihood Estimator (MLE), whereas the temporal autocorrelation of the residuals was modelled using an order 1 autoregressive model AR(1)^47^. We simulated and added *OD* changes to real, resting-state data at different SNR levels, then evaluated the efficiency of MLE to deconvolve the shape of different simulated HRFs after reconstruction along the cortical surface. Our deconvolution approach was finally illustrated using fNIRS data acquired from subjects during motor or visual tasks and data from spontaneous epileptic activity in one epilepsy patient.

## Material and methods

### Optimal montages for personalized fNIRS investigations

The “optimal montage” methodology originally proposed in Machado et al.^15^ and further improved in Machado et al.^18^ consists in estimating the optimal positions of a specific number of sources and detectors along the scalp surface, providing the best sensitivity to a target brain volume identified a priori. The procedure consists of the following steps: (1) Defining the set of possible optode positions along the high density tessellated surface of the scalp ($\sim$ 4000 vertices, edge length $\sim$ 5 mm) segmented from the anatomical T1-MRI of the subject^48^. (2) Performing Monte Carlo simulations for photon transport into biological tissues^49,50^ in order to generate “all” possible sensitivity profiles ($\sim$ 200,000) considering only source-detector separations likely to provide good SNR data. (3) Solving a mixed linear integer problem given specific functional constraints such as optode density (minimum optode-optode separation = 2 cm), the minimal/maximal source-detector separations (between 2.5 and 3.5 cm) and pair density (each source being measured by at least two detectors within the separation constrains).

### fNIRS forward model

**Figure 1:**
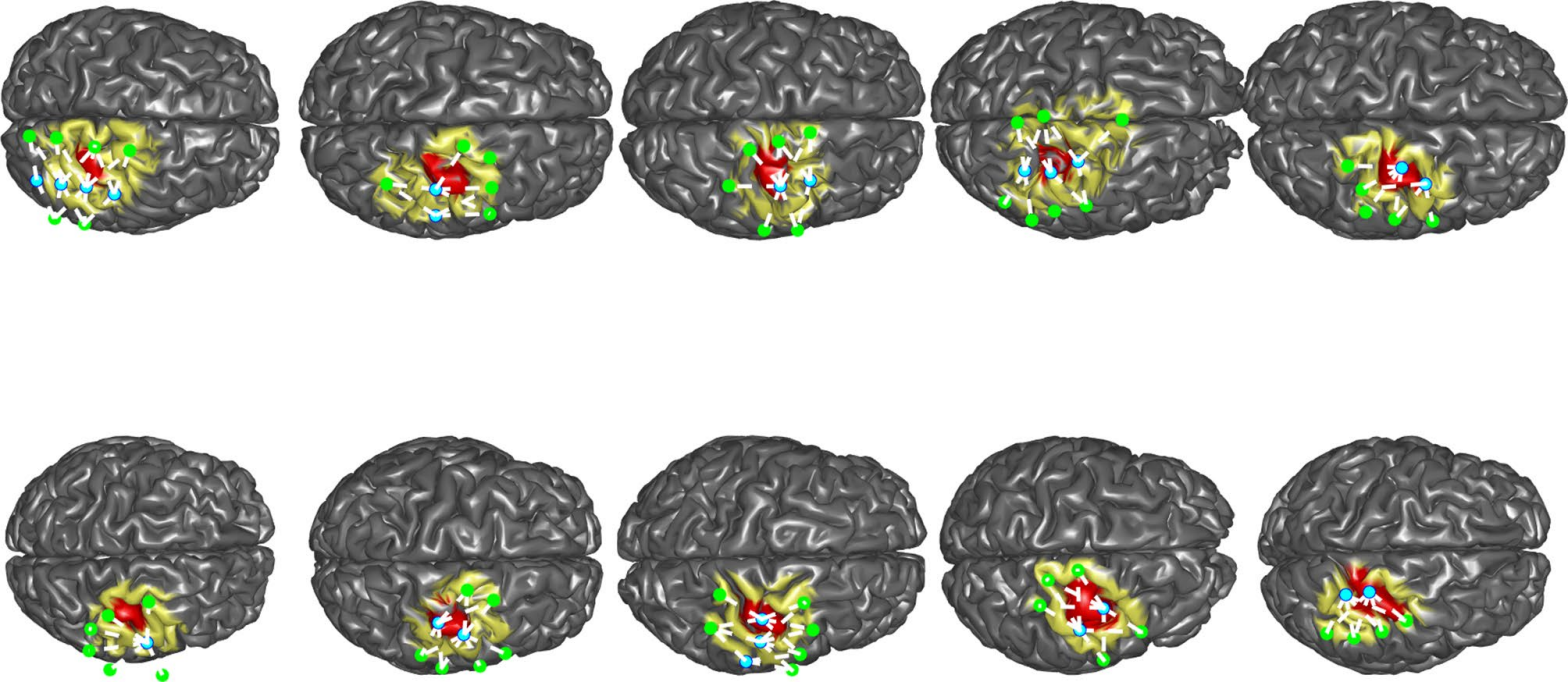
Representation of the 10 anatomical models considered for realistic simulations: sources of the optimal montage are represented in blue, detectors are represented in green. The white line represent the different pairs of the montage. The montage FOV (vertices where the sensitivity matrix $>10\%$ of max) is represented in yellow on the cortical surface. The right hand knob area manually segmented to define a target for the optimal montage is represented in red.

For a specific set of source-detector positions along the scalp, the fNIRS forward model describes how local variations in absorption coefficients $\Delta \mu _a$, mainly due to local fluctuations of [*HbO*] and [*HbR*], impact $\Delta OD$ measurements on the scalp. Considering all source-detector measurements, the fNIRS forward model at wavelength $\lambda$ is thus described by the following linear model:

1 $$\begin{aligned} \varvec{\Delta OD}^{\lambda }=\varvec{A}^{\lambda } \varvec{\Delta \mu _a}^{\lambda }. \end{aligned}$$

where matrix $\varvec{\Delta OD}^{\lambda }$ denotes the change in optical density (number of measurements $\times$ number of time samples, measured in log base e) and $\varvec{\Delta \mu _a}^{\lambda }$ (number of vertices $\times$ number of time samples) represents the distribution of absorption changes along the cortical surface to be estimated. $\varvec{A}^{\lambda }$ (number of measurements $\times$ number of vertices on the cortical surface) is the surfacic sensitivity matrix built from 3D Monte Carlo simulations of infrared light transport within the head tissues^49,50^. The volumetric sensitivity matrix obtained after Monte Carlo simulations was indeed constrained in the gray matter and projected on the cortex surface mesh (i.e., pial surface). To do so, we used the volume-to-surface interpolation method proposed in Grova et al.^51^, allowing to preserve sulco-gyral morphology. The surfacic sensitivity matrix was then thresholded at 10% of its maximum coefficient value (see Fig. 1) thus defining the montage Field of View (FOV) providing the largest sensitivity to underlying cortical areas.

### Inverse modelling using restricted maximum likelihood (ReML) method

Reconstruction of $\Delta [HbO]$ and $\Delta [HbR]$ along the cortical surface was performed using the Restricted Maximum Likelihood method^52^. The inverse problem was formulated as the following two-level hierarchical, fusion model for both wavelengths:

2 $$\begin{aligned} \left[ \begin{array}{c} \varvec{\Delta OD}^{685} \\ \varvec{\Delta OD}^{830} \end{array} \right]&= \begin{bmatrix} \varvec{A}^{685} &{} 0 \\ 0 &{} \varvec{A}^{830} \end{bmatrix} ~ \left[ \begin{array}{c} \varvec{\Delta \mu _a}^{685} \\ \varvec{\Delta \mu _a}^{830} \end{array} \right] + \begin{bmatrix} \varvec{E_1}^{685} &{} 0 \\ 0 &{} \varvec{E_1}^{830} \end{bmatrix}\end{aligned}$$

3 $$\begin{aligned} \left[ \begin{array}{c} \varvec{\Delta \mu _a}^{685} \\ \varvec{\Delta \mu _a}^{830} \end{array} \right]&= \left[ \begin{array}{c} \varvec{0}\\ \varvec{0} \end{array} \right] + \begin{bmatrix} \varvec{E_2}^{685} &{} 0 \\ 0 &{} \varvec{E_2}^{830} \end{bmatrix}. \end{aligned}$$

where $\varvec{E_1}^{\lambda }$ represents the measurement noise and is modelled as multivariate normal distribution $\mathcal {N}^P(\varvec{0}_P, h_1~\varvec{C_1}^{\lambda })$, *P* being the number of channels and the scalar $h_1$ being an hyperparameter which acts as a weighting factor for the spatial covariance matrix $\varvec{C_1}^{\lambda }$. Similarly, $\varvec{E_2}^{\lambda } \sim \mathcal {N}^V(\varvec{0}_V, h_2~\varvec{C_2}^{\lambda })$ represents the multivariate a priori distribution of the absorption changes along the cortex, *V* being the number of vertices. In this study, we estimated $\varvec{C_1}^{\lambda }$ from the $\varvec{\Delta }\mathbf{OD} ^{\lambda }$ measurements within the initial rest period while $\varvec{C_2}^{\lambda }$ was modelled as the identity matrix, which is equivalent to a L2-minimum norm estimate approach^53^. Estimation was performed using restricted maximum likelihood which iteratively calculates the parameters and hyperparameters of the model until convergence of the free energy of the model^54^. Finally, relative changes in hemoglobin along the cortical surface were obtained from the absorption coefficients as follows:

4 $$\begin{aligned} \left[ \begin{array}{c} \Delta [HbR] \\ \Delta [HbO] \end{array} \right]&= \begin{bmatrix} \alpha _{HbR}^{685} &{} \alpha _{HbO}^{685} \\ \alpha _{HbR}^{830} &{} \alpha _{HbO}^{830} \end{bmatrix}^{-1} ~ \left[ \begin{array}{c} \Delta \mu _a^{685} \\ \Delta \mu _a^{830} \end{array} \right] \end{aligned}$$

where $\alpha ^{\lambda }_{HbX}$ are the specific molar absorption coefficient in [$\hbox {cm}^{-1}\,\mathrm{Molar}^{-1}$] (tabulated data available at https://omlc.org/spectra/hemoglobin/summary.html).

### HRF estimation

The deconvolution problem can be treated within a GLM framework, modelling the unknown HRF $\mathbf{h} =[h[i],1 \le i \le K]$ as a linear combination of *K* delta functions ($\delta$ such that $\delta [x-a]=0$ for $x\ne a$) with amplitude $\beta _i$. Let $[k_0 \quad k_1]$ be the pre-post stimulus range for the HRF expressed in time samples such that the total number of samples $K=k_1-k_0+1$. Note that because the HRF may start before the stimulus, we may have ($k_0<0$)^55,56^. This model is known as the Finite Impulse Response (FIR) basis set^42^:

5 $$\begin{aligned} h[i]=\sum _{k=k_0}^{k_1} \delta [i-(k-k_0+1)] \beta _{k-k_0+1}, \quad 1 \le i \le K \end{aligned}$$

The GLM can then be expressed as follows:

6 $$\begin{aligned} \mathbf{y}&=\mathbf{s} * \mathbf{h} + confounds + errors \end{aligned}$$

7 $$\begin{aligned} y[n]&=\Big [\sum _{i=1}^{K} s[n-(i+k_0-1)] \beta _i \Big ] + \sum _{m=1}^{M} d_m[n]\beta _{K+m} + e[n], \quad 0 \le n \le N-1 \end{aligned}$$

where $\mathbf{y} =[y[n],0 \le n \le N-1]$ represents either $\Delta [HbO]$ or $\Delta [HbR]$ signals reconstructed on a specific vertex along the cortical surface at time sampling index *n* and $\mathbf{s} =[s[n],0 \le n \le N-1]$ represents the stimulus (usually a binary vector encoding the occurrence of events or epochs). $*$ represents the convolution operator. For the equation to be valid close to borders, we must assume that $s[n]=0$ for $n>N-1$ and $n<0$. To simplify, we considered a single stimulus but the model can be extended easily to multiple stimuli. The model also accounts for some drifts $d_m[n]$ modelled using *M* cosines functions (< 0.01 Hz) from the discrete cosine transform basis set^57^ and random additive noise *e*[*n*].

8 $$\begin{aligned} d_m[n]&=\sqrt{(}1/N)~\text {for}~m=1 \end{aligned}$$

9 $$\begin{aligned} d_m[n]&=\sqrt{(}2/N)cos(\frac{\pi (2n+1)(m-1)}{2N})~\text {for}~2\le m \le M \end{aligned}$$

Considering all sampling indices together, the matrix formulation of the problem leads to the following equation:

10 $$\begin{aligned} \mathbf{y} = \mathbf{X} \varvec{\beta }+ \mathbf{e} \end{aligned}$$

with $\mathbf{y} =[y[n],0 \le n \le N-1]$ the column vector of observed data ($N=3000$ samples in this study). $\mathbf{X}$ is the design matrix consisting of $L=K+M$ covariates. The first *K* columns of $\mathbf{X}$ are the shifted versions of the binary stimulus vector (i.e., Toeplitz matrix) while the *M* last columns represent the set of cosine functions modelling the drifts. $\varvec{\beta }$ is the vector of *L* regression coefficients we wish to estimate, and $\mathbf{e} =[e[n],0 \le n \le N-1]$ is a vector of unexplained error values. $\mathbf{e}$ is assumed to follow a *N* dimensional multivariate normal distribution $\mathbf{e} \sim \mathcal {N}^{N}(\mathbf{0} _N,\mathbf{V} )$ where $\mathbf{V} =\sigma ^2\varvec{\Omega }$ is the $N \times N$ positive-definite variance covariance matrix, $\varvec{\Omega }$ being the autocorrelation structure of the noise $\mathbf{e}$ and $\sigma ^2$ a scaling factor.

### Maximum likelihood estimator with non spherical disturbance

In order to estimate $\varvec{\beta }$ and $\sigma ^2$, the generalized regression problem presented in Eq. 10 can be solved using a maximum likelihood approach^58^. Assuming $\varvec{\Omega }$ to be known (see “Autoregressive model for Ω” section), the Maximum Likelihood Estimator (MLE) is given by:

11 $$\begin{aligned} \hat{\varvec{\beta }}&=(\mathbf{X} ^T\varvec{\Omega }^{-1}{} \mathbf{X} )^{-1}{} \mathbf{X} ^T\varvec{\Omega }^{-1}{} \mathbf{y} \end{aligned}$$

12 $$\begin{aligned} \hat{\sigma }^2&=\frac{1}{N}(\mathbf{y} -\mathbf{X} \hat{\varvec{\beta }})^t\varvec{\Omega }^{-1}(\mathbf{y} -\mathbf{X} \hat{\varvec{\beta }}) \end{aligned}$$

$\hat{\varvec{\beta }}$ is the best linear unbiased estimator of $\varvec{\beta }$ following a L-multivariate normal distribution $\hat{\varvec{\beta }}\sim \mathcal {N}^L(\varvec{\beta },\sigma ^2(\mathbf{X} ^T\varvec{\Omega }^{-1}{} \mathbf{X} )^{-1})$ while $\hat{\sigma }^2$ is an estimator following a central chi-squared univariate distribution $\mathcal {\chi }^2$ with $N-L$ degree of freedom, $\hat{\sigma }^2 \sim \frac{\sigma ^2}{N} \mathcal {\chi }^2_{N-L}$.

### Partial F-test for a subset of $\beta$ coefficients

A question of interest is whether the estimated HRF coefficients (i.e., $\hat{\mathbf{h }}=[\hat{\beta _1},\ldots ,\hat{\beta _K]}$) are significantly different from zero. Let $\varvec{\beta }_h$ be the vector that consists of the subset of $\varvec{\beta }$ excluding the drift terms (therefore considering only the *K* first values, hence the name partial). We tested the following null hypothesis:

13 $$\begin{aligned} H_0:\beta _{1} = 0 \quad \text {and} \quad \beta _{2} = 0, \ldots \quad \text {and} \quad \beta _{K} = 0 \end{aligned}$$

against the alternative hypothesis

14 $$\begin{aligned} H1:\beta _{1} \ne 0 \quad \text {or} \quad \beta _{2} \ne 0, \ldots \quad \text {or} \quad \beta _{K} \ne 0 \end{aligned}$$

Let $\Theta _h$ be a $K \times K$ submatrix (first K rows and columns) of $(\mathbf{X} ^t\varvec{\Omega }^{-1}{} \mathbf{X} )^{-1}$. Replacing $\sigma ^2$ by its estimator $\hat{\sigma }^2$ in the variance-covariance expression of $\hat{\varvec{\beta }}$, it can be demonstrated^59^ that the resulting *F* statistic is following, under the $H_0$ hypothesis, a univariate Fisher’s probability density function with *K* and $N-L$ degrees of freedom:

15 $$\begin{aligned} F=\frac{N-L}{N}\frac{\hat{\varvec{\beta }}_h^T \Theta _h^{-1} \hat{\varvec{\beta }}_h}{K\hat{\sigma }^2} \sim \mathcal {F}_{K,N-L} \end{aligned}$$

The null hypothesis $H_0$ is rejected with a significance level of 5% if $F>F_{th}$, where $F_{th}$ is the upper bound of the Fisher’s probability density function.

### Autoregressive model for $\varvec{\Omega }$

As suggested in fMRI^47,60^ and fNIRS^21–23,61^, the temporal autocorrelation of the error term in 10, i.e. $\varvec{\Omega }$, can be modelled using an AutoRegressive model of order 1 (AR(1)). To do so, a preliminary maximum likelihood estimation is typically conducted assuming $\varvec{\Omega }= \mathbf{I} _N$. $\hat{\varvec{\Omega }}$ is then estimated from the residual vector solving the Yule Walker equations for the autoregression coefficient. A second analysis is finally conducted using the estimator $\hat{\varvec{\Omega }}$ in Eq. 11 and 12.

### Specificity of the model when applied to real fNIRS data

Because the proposed model relies heavily on the assumptions regarding the error term structure, the specificity of the proposed AR(1)-MLE and partial F-based inference was evaluated in a separate study, and reported in supplementary material 1. Our objective was to control that our proposed statistic follows its theoretical F distribution under the null hypothesis when applied to serially correlated fNIRS data. To do so, a surrogate dataset consisting of $\Delta$[HbO], $\Delta$[HbR] runs acquired at rest and resampled in the time-frequency domain^62^ was used. We found for both HbO and HbR signals and different paradigms that the empirical false positive rate was very close to its expected value of 5% using AR(1)-MLE modeling (cf. supplementary material 1).

## Validation procedure

### Subjects selection and pre-processing

We retrospectively selected from our database fNIRS data from 10 healthy participants (mean age 25) with no history of neurological or psychiatric disorders. For every subjects, we applied a personalized optimal montage (maximum 4 sources and 8 detectors) targeting specifically the right motor cortex of every subject. The right hand knob region of every subject was visually identified and marked on the cortical surface by an expert anatomist (GP). This motor region will be further denoted as our target Region of Interest (ROI) (cf. Fig. 1). We used a neuronavigation device to localize the sensors position on the head of the subjects and used collodion to glue the sensors which optimize the optical coupling^16,18,63^. Data were acquired at rest with subjects sitting in a comfortable chair according to our fNIRS protocol, approved by the research ethics committee of the Montreal Neurological Institute and the McConnell Brain Imaging Center. Methods were carried out in accordance with relevant guidelines and regulations. Informed consent was obtained from all participants and/or their legal guardians. Runs were acquired for 10 min (N = 3000 samples, sampling rate 5 Hz) with the Brainsight CW fNIRS system (685 and 830 nm). For all selected participants, we also acquired an anatomical T1-weighted MRI acquisitions (Siemens MAGNETOM 3T, TR = 2.3 s, TE = 2.98 ms, flip angle = 9 degrees, voxel resolution $1~\text {mm}^3$) for anatomical head modelling^48^.

After acquisitions, fNIRS runs were reviewed to exclude large movement artefacts. Small movement artefacts were marked manually and corrected using a spline interpolation subtraction technique^64^. All selected pairs were then high pass filtered (high cut-off 0.005 Hz) to remove very slow trends. Detrending the data was necessary to define a stable baseline necessary to convert measured intensity signals into $\Delta [HbO]$ and $\Delta [HbR]$ using the modified Beer Lambert Law. Selected data were considered to evaluate the proposed deconvolution method.

#### Simulation of realistic fNIRS measurements

For each vertex $\nu$ of the ROI (red patch), we generated simulations by assuming an homogeneous change of hemoglobin concentration, denoted as $\Delta [HbO]_{roi}$ and $\Delta [HbR]_{roi}$. The time course of these concentration changes was estimated by a convolution of a binary stimulation vector and a simulated HRF. For this evaluation, we considered a rapid event related paradigm (30 trials of 200 ms, inter-trial-interval (ITI): 2–60 s randomly distributed) and we simulated 4 HRF models, all generated by a linear combination of 2 Gamma functions^65^ (see detailed parameters in Table 1).

16 $$\begin{aligned} HRF(t)&= \left( \frac{t}{TTP1}\right) ^{a_1} e^{-\left( \frac{t-TTP1}{b_1}\right) } - \gamma \left( \frac{t}{TTP2}\right) ^{a_2} e^{-\left( \frac{t-TTP2}{b_2}\right) } \end{aligned}$$

17 $$\begin{aligned} ~\text {with}~a_i&=8log(2)\frac{TTPi^2}{FWHMi^2}, \quad b_i=\frac{FWHMi^2}{8log(2) TTPi}, \quad i=1,2 \end{aligned}$$

**Table 1:** Parameters for the HbO simulated *HRFs*.

Parameters	TTP1 (s)	FWHM1 (s)	TTP2 (s)	FWHM2 (s)	$\gamma$
HRF1 SPM canonical	6	5.2	15	9	0.1
HRF2 short duration	5	1	6	2	0.1
HRF3 long duration	5	10	–	–	0
HRF4 large undershoot	5	5.2	15	9	0.5

*TTP* time to peak, *FWHM* full width at half maximum.

**Figure 2:**
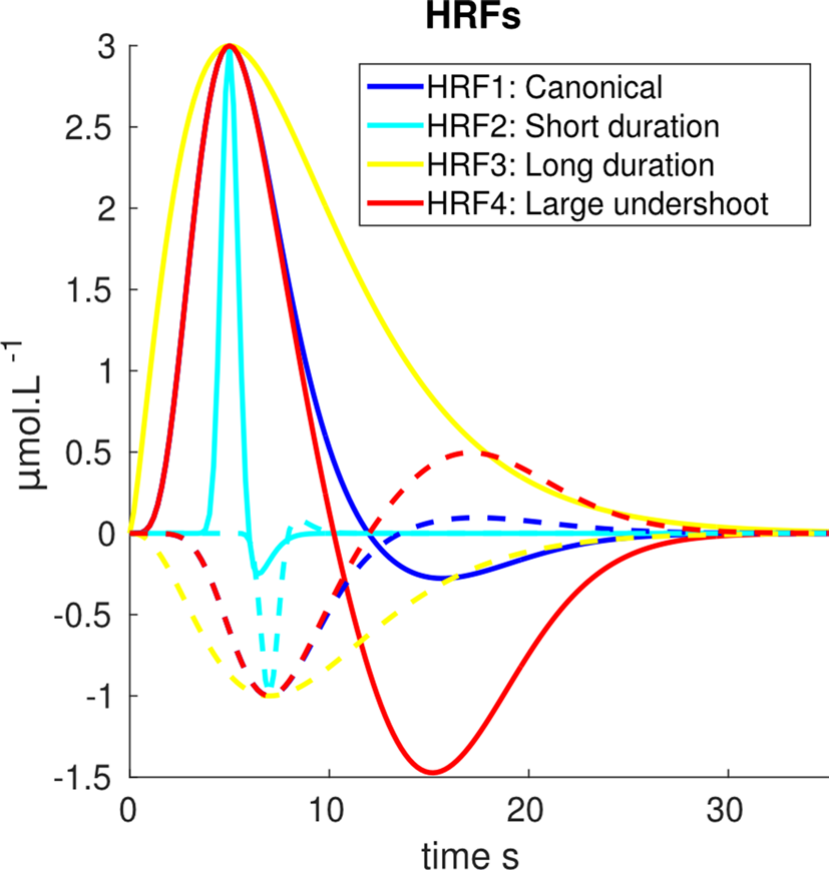
Simulated HRF models for HbO (plain) and HbR (dashed).

The same parameters were used to model HbO versus HbR HRFs, except for the peak latencies (TTP1 and TTP2) that were delayed by 2 s for HbR, as suggested by Jasdzewski et al., Wolf et al.^35,66^. The four different HRFs were scaled such that maximal amplitude was $3\,\upmu \,\mathrm{mol}^{-1}$ for HbO and $-1\,\upmu \,\mathrm{mol}^{-1}$ for HbR (see Fig. 2).

The homogeneous hemoblobin concentration changes in the ROI were then converted into $\varvec{\Delta \mu _{a,roi}}^{830}$ and $\varvec{\Delta \mu _{a,roi}}^{685}$ by spectral decomposition of the extinction coefficients. To generate noise-free evoked $\varvec{\Delta OD^{830}}_{noiseFree}$ and $\varvec{\Delta OD}^{685}_{noiseFree}$ for a given montage, we applied the forward model equation (cf. Eq. 1) to local changes in absorption. Finally, in order to model realistic physiological fluctuation and instrumental noise, real resting state data acquired for each subject (i.e.,$\varvec{\Delta OD}^{830}_{rest}$ and $\varvec{\Delta OD}^{685}_{rest}$) were scaled by a global factor $\tau$ (see Eq. 20) and added to the simulated noise free changes (Fig. 2) as suggested in Brigadoi et al.^46^.

18 $$\begin{aligned} \varvec{\Delta OD}^{\lambda }_{sim}&=\varvec{\Delta OD}^{\lambda }_{noiseFree}+\tau \varvec{\Delta OD}^{\lambda }_{rest} \end{aligned}$$

19 $$\begin{aligned} \text {where} ~ \varvec{\Delta OD}^{\lambda }_{noiseFree}&=\varvec{A}^{\lambda } \varvec{\Delta \mu _{a,roi}}^{\lambda }. \end{aligned}$$

**Figure 3:**
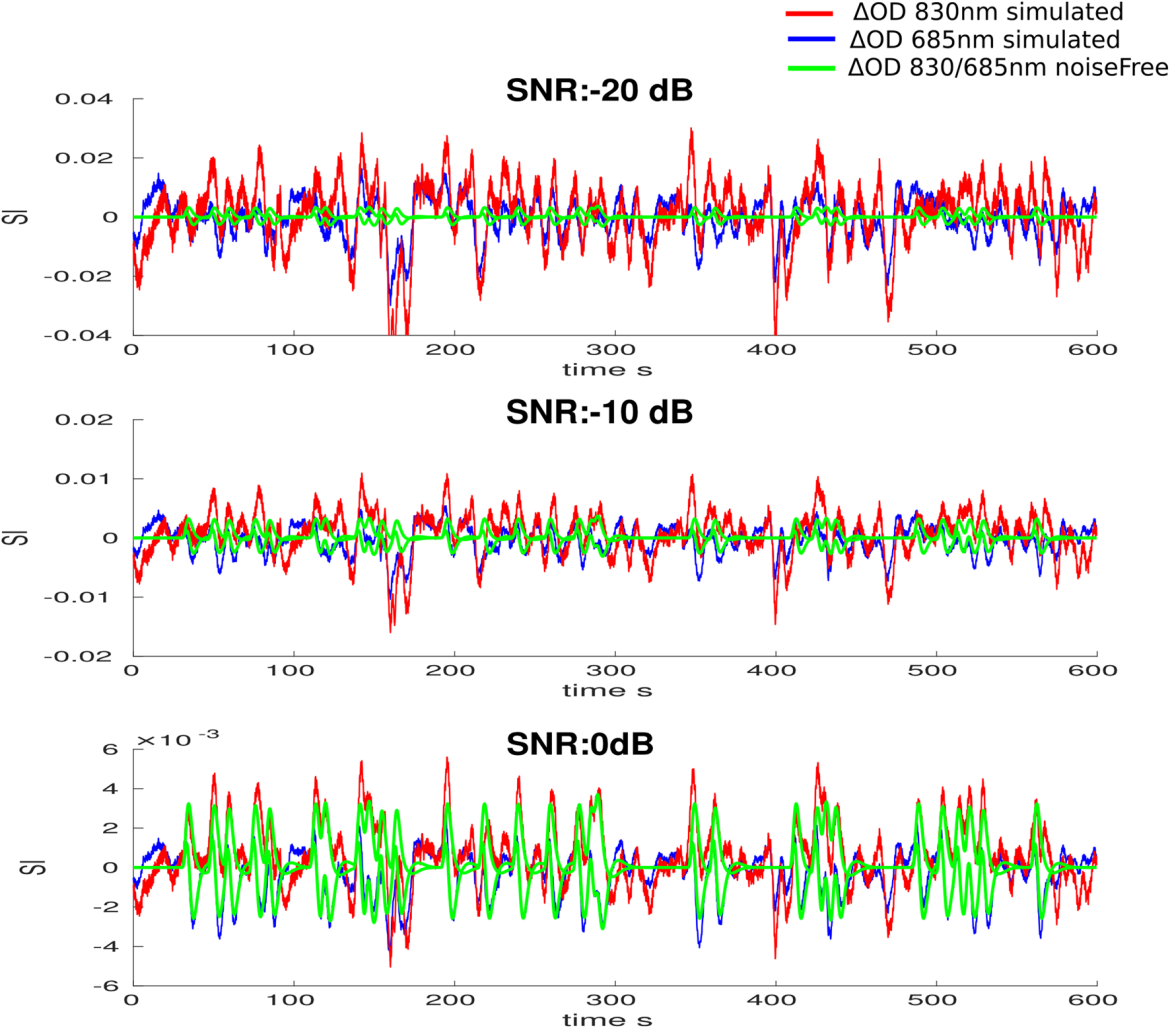
Realistic simulations of fNIRS data: $\Delta OD_{sim}$ at 830 nm (red) and 685 nm (blue) simulated at different SNR levels on the scalp surface of one anatomical model and for the most sensitive pair to the target ROI. Noise free simulated $\Delta OD$ are represented in green for both wavelengths.

The same scaling factor $\tau$ was applied to all pairs of the montage at both wavelengths so that all relative amplitudes were preserved. $\tau$ was tuned to model the expected SNR of our simulations, defined as follows, using $\Delta OD^{830}$ measurements from the most sensitive pair over the target hand knob area:

20 $$\begin{aligned} SNR=10log_{10}\left( \frac{<\Delta OD^{830}_{roi}>}{\tau ^2 <\Delta OD^{830}_{rest}>}\right) \end{aligned}$$

where operator $<X>$ denotes the signal power computed as the mean sum of squares. We simulated different SNR levels ranging from −30 to 10 dB by steps of 2 dB. Figure 3 illustrates some simulated $\Delta OD_{sim}$ signals at different SNR levels.

#### HRF deconvolution applied on local DOT reconstruction

DOT reconstruction of $\Delta [HbO]$ and $\Delta [HbR]$ along the cortical surface from $\Delta OD^{830}_{sim}$ and $\Delta OD^{685}_{sim}$ was performed using the Restricted Maximum Likelihood method (see “Inverse modelling using restricted maximum likelihood (ReML) method” section). Deconvolution using AR(1)-MLE was then applied to signals of each vertex $\nu$ in the montage specific FOV as described in “Maximum likelihood estimator with non spherical disturbance” section. The resulting statistical parametric F-maps were thresholded at a significance level of 5% after applying correction for multiple comparison with Bonferonni correction using the total number of vertices within the FOV.

#### Validation metrics

The accuracy of the deconvolution was first evaluated at the vertex $\nu _{m}$ along the cortical surface exhibiting the maximum coefficient $|\hat{\beta _i}|$ at sampling index *i* corresponding to the first peak of the estimated HRF (i.e., amplitude of TTP1). To assess the accuracy of the shape of the reconstructed HRF independently from the quantitative accuracy on its amplitude, we first normalized the reconstructed HRF to a maximum of 1. We estimated the shape error of the estimated HRF as the Mean Squared Error (MSE) between $\hat{\mathbf{h }}(\nu _{m})$ and the corresponding simulated HRF. We also evaluated the errors made on the estimation of the delays and durations of the main peak (TTP1, FWHM1) and of the undershoot (TTP2, FWHM2). To do so, the normalised $\hat{\mathbf{h }}(\nu _{m})$ was fitted with the difference of two gamma density functions. Five parameters (TTP1, FWHM1, TTP2, FWHM2, $\gamma$) were optimized simultaneously using a nonlinear least-squares fitting algorithm^65^, using the true simulated parameters of each HRF as initial conditions (see Table 1).

## HRF deconvolution applied on DOT reconstruction for real acquisitions

### fNIRS response to a motor task

We analyzed fNIRS data (10 min, acquired at 5 Hz) during a left hand finger opposition task using a personalized optimal montage positioned over the right hand knob area for two right handed subjects. Methods were carried out in accordance with relevant guidelines and regulations. Informed consent was obtained from all participants. The event related paradigm consisted of 30 trials followed by 2–60 s of rest. Participants were instructed by an auditory cue to press their thumb and index fingers for 1 s against each other then relax. Data were band pass filtered [0.005 0.6] Hz and converted into $\Delta OD$. DOT was applied to reconstruct $\Delta [HbO]$ and $\Delta [HbR]$ along the cortical surface, before applying deconvolution for each vertex $\nu$ of the montage FOV. In order to get an idea of the overall signal quality recorded on the scalp surface, we estimated an SNR index using the same formalism as in simulations. After selecting the most sensitive $\Delta OD$ channel at 830 nm to the target brain region, we performed a deconvolution to predict what is the approximate noise free $\Delta OD$ response at the sensor level. SNR was then estimated using the logarithmic ratio of the predicted response to the residuals.

### fNIRS response to a visual task

We also analyzed fNIRS data (10 min, acquired at 5 Hz) during a visual task using a personalized optimal montage positioned over the right visual cortex (V1) for one right handed subject. The visual task was a left hemi-field black and white checkerboard flashing at 5 Hz. The event related paradigm consisted of 50 trials of 2.4 s duration followed by followed by 5–15 s of rest. Data were analyzed using the same methodology considered for the motor task.

#### fNIRS response to transient epileptic discharges

From our previous EEG/fNIRS study^16^, we selected one run of 15 min from one patient with focal epilepsy who underwent simultaneous EEG/fNIRS acquisitions during 4 h. The selected patient was a 24 years old female diagnosed with right frontal epilepsy. EEG/MEG source localization suggested a right frontal generator for the epileptic discharges. EEG was acquired using 25 scalp electrodes placed according to the 10–20 system. fNIRS montage was tailored using the optimal montage methodology to target a right frontal epileptic focus, identified using MEG source localization. The montage was then completed to cover also the homologous contralateral region to the presumed focus. Epileptiform discharges consisted of 10 bursts of rhythmic fast activity in the beta range (20–25 Hz) lasting 0.5–1 s each. DOT was applied on the $\Delta OD$ time series to reconstruct $\Delta [HbO]$ and $\Delta [HbR]$ along the cortical surface. A deconvolution analysis was then performed to reconstruct the HRF associated with these bursts with the same parameters as described in “Maximum likelihood estimator with non spherical disturbance” section.

## Results

### HRF deconvolution applied on local DOT reconstruction using realistic simulations

**Figure 4:**
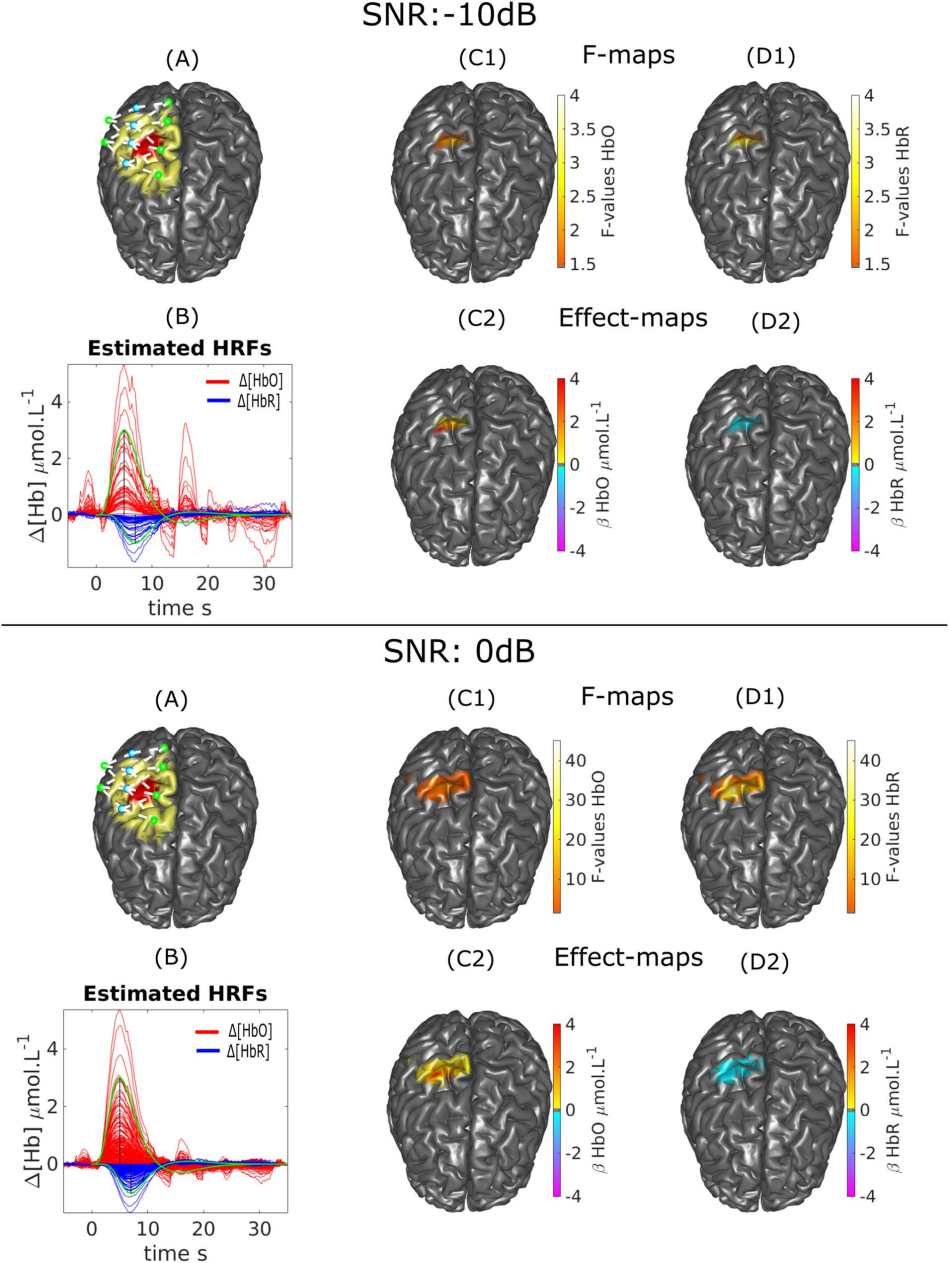
HRF deconvolution applied on DOT reconstruction on realistic simulations from one anatomical model obtained at two SNR levels (− 10 dB and 0 dB) (**A**) Optimal montage, simulated ROI (red) and FOV (yellow). (**B**) Estimated HRF time courses. HRF1 theoretical models simulated for either HbO/HbR are represented in green. (**C1**, **C2**) $\Delta [HbO]$: Thresholded F-map ($\alpha =0.05$, Bonferronni corrected) and associated effect size map estimated at $TTP1=6$ s. (**D1**, **D2**) $\Delta [HbR]$: Thresholded F-map ($\alpha =0.05$, Bonferronni corrected) and associated effect size map estimated at $TTP1=8$ s.

Figure 4 illustrates one of the selected anatomical models, the corresponding F map and the full time courses of the reconstructed HRF for all the vertices of the FOV passing the significance threshold according to the F-test. We are also representing the effect size map for the coefficient $\hat{\beta _i}$ of the estimated HRF corresponding to the first peak TTP1 (6 s for HbO and 8 s or HbR). At a SNR of 0 dB, the peak of the effect size map was well localized in the simulated ROI for both HbR and HbO, whereas the spatial extent was slightly overestimated (see F maps). AR(1)-MLE was able to reconstruct the simulated HRFs quite accurately for all vertices of the F map. When considering a lower SNR of $-10$ dB, the peak of the effect size was still well localized in the ROI, whereas the spatial extent was underestimated (see F maps). The estimation of the temporal time courses for both HbO and HbR were quite accurate even in noisier conditions. However, strong physiological fluctuations were captured when the expected response was of low amplitude, i.e. between − 5 and 0 s and during the undershoot period. For all the 10 subjects anatomical models, similar spatial and temporal reconstructions patterns were observed for these two SNR levels.

**Figure 5:**
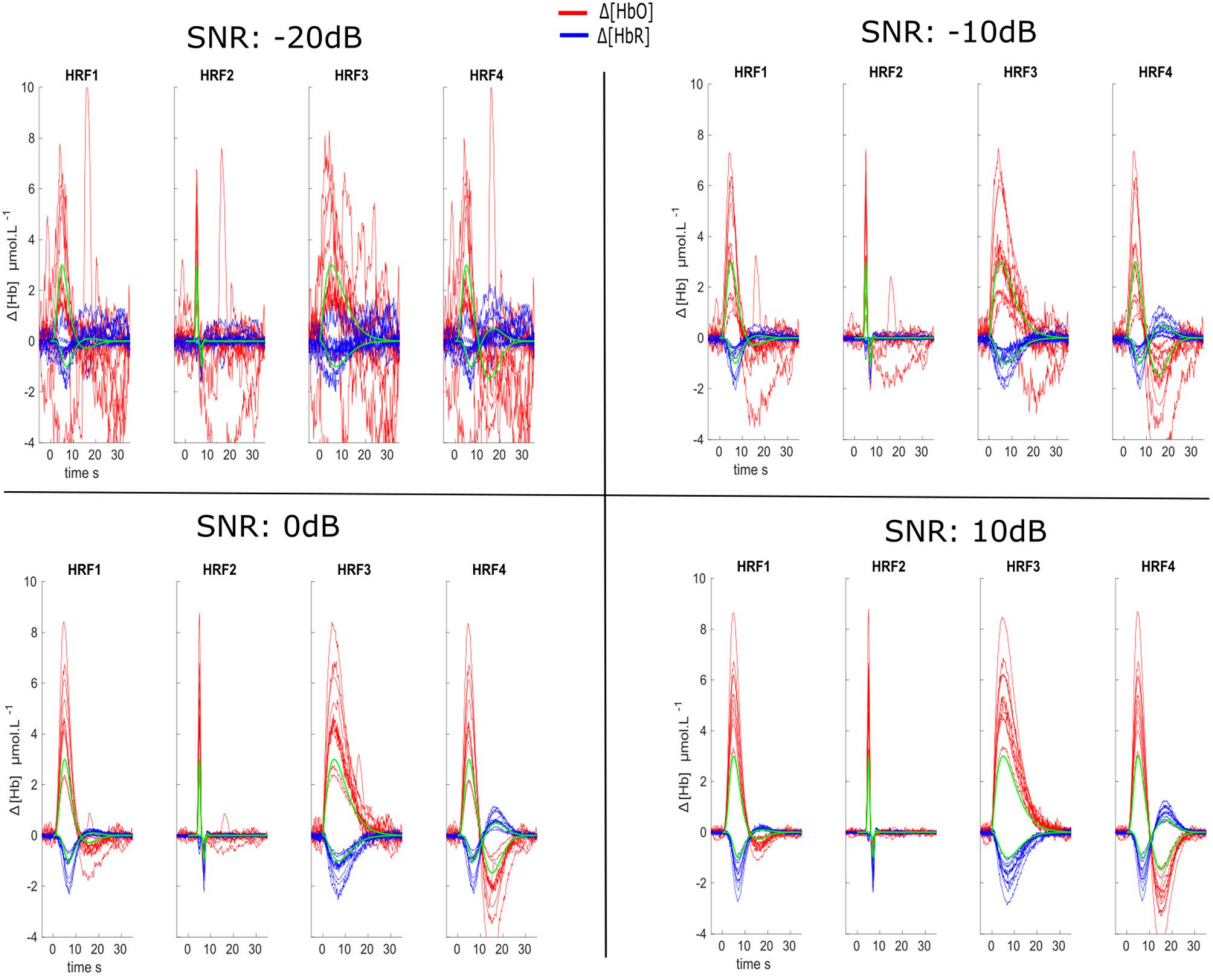
Reconstructed HRF time courses for four different SNRs from the 10 anatomical models and at the vertex along the cortical surface with maximal amplitude at TTP1. Red–Blue lines represents different anatomical models. The simulated HRFs (i.e., ground truth) are shown in green.

Figure 5 shows the estimated HRF obtained at the significant vertex exhibiting the maximal activity at TTP1 for the realistic simulations obtained from all 10 anatomical models. Note that at − 20 dB and below, no reconstructions were found statistically significant according to Fisher’s test, so the estimated HRFs are presented for comparison purpose only. At − 20 dB, the HRF estimations were relatively poor, highly contaminated with physiological fluctuations within the whole estimation window. The results at − 10 dB were actually quite accurate, for all of the 4 simulated HRF shapes, especially along the first peak of the response, while the estimated time course was noisier during the undershoot. At higher SNRs (0 and 10 dB) the reconstruction accuracy improved significantly.

**Figure 6:**
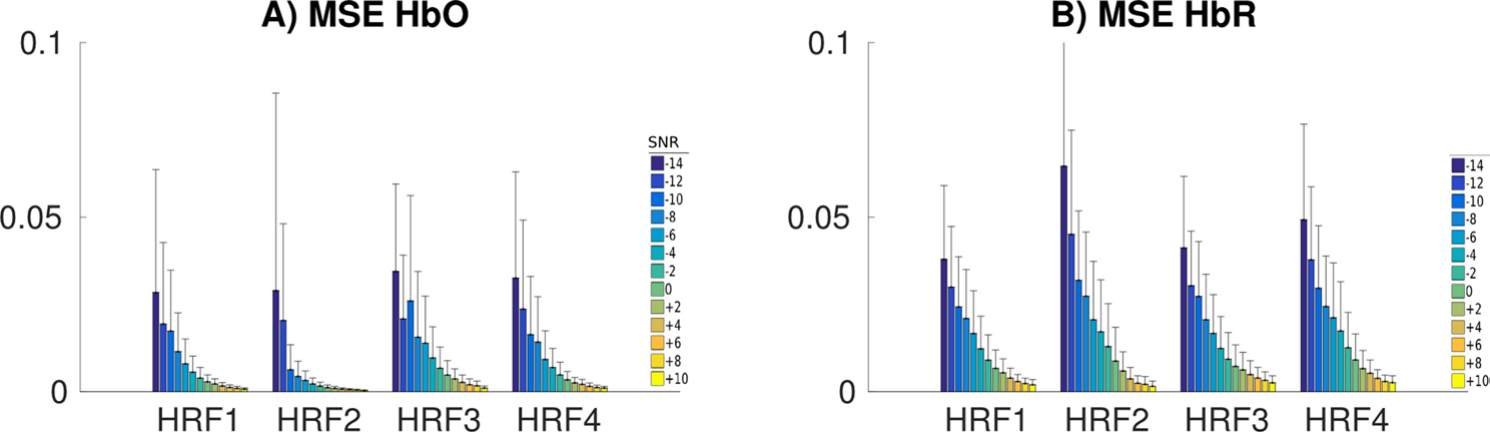
Distributions of MSE scores as a function of the SNR level for different HRF models. We show only the scores for SNRs ranging from − 14 dB (poor estimation below − 14 dB) to + 10 dB by steps of + 2 dB.

Figure 6 shows the accuracy of the reconstructed time course of the HRF, as assessed by mean MSE and corresponding standard deviation obtained over the 10 anatomical models. Our results are suggesting that the accuracy of HRF estimations improved substantially when increasing the SNR for all different HRF models. At a given SNR level, MSE was slightly lower for HbO when compared to HbR, as we simulated $\Delta [HbR]$ changes with lower amplitude than $\Delta [HbO]$.

**Figure 7:**
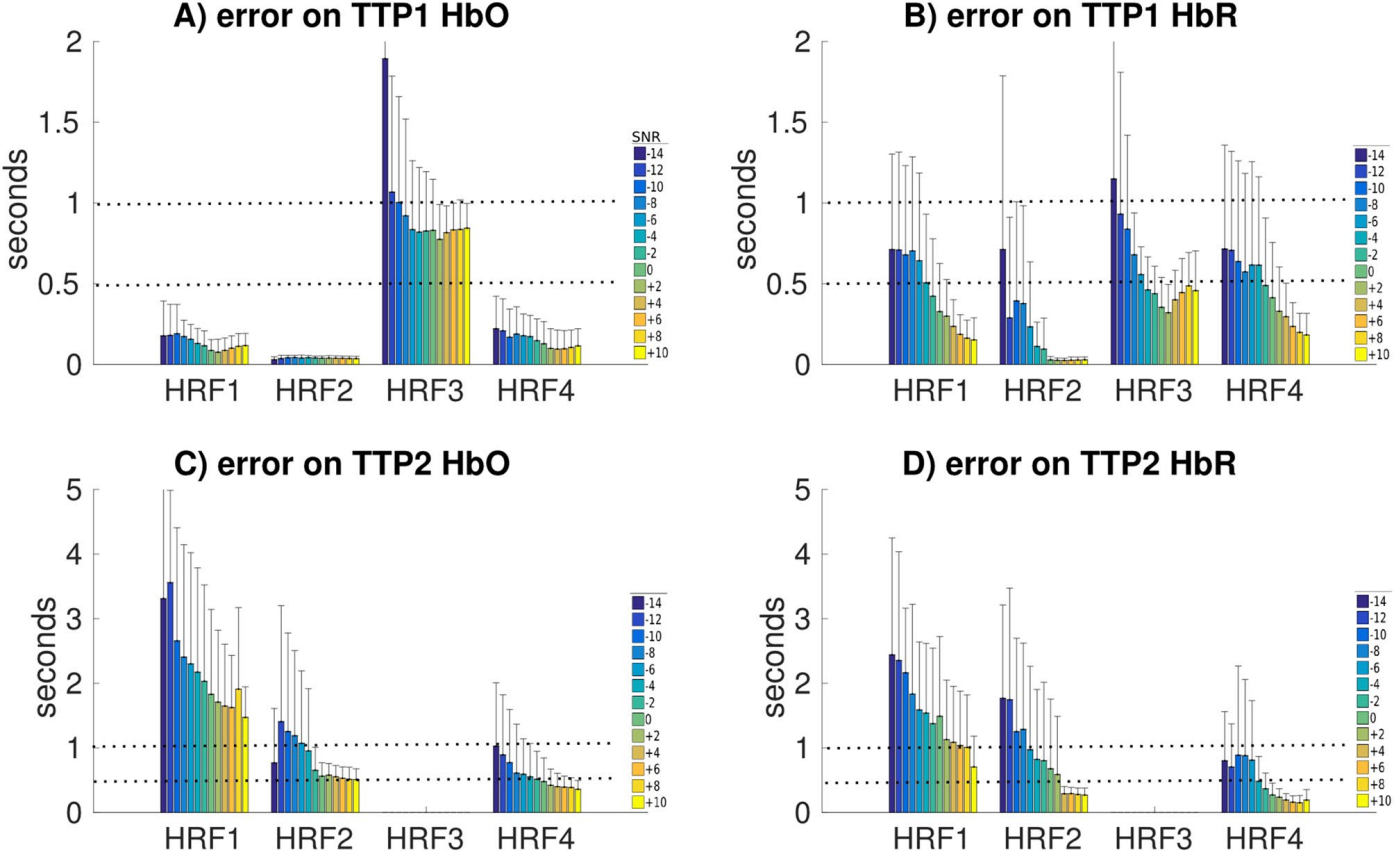
Distributions of the errors in the estimation of TTP as a function of the SNR level for all HRF models. Note that TTP2 for HRF3 was not estimated as no undershoot were simulated.

Errors in estimation of the first peak of the HRF (TTP1) for all HRF models investigated are reported in Fig. 7 A for HbO and Fig. 7 B for HbR. For HbO deconvolution, estimation of the first peak was quite accurate (mean error below 0.25 s) for HRF models 1, 2 and 4. Corresponding errors for HbR were larger but still quite accurate and decreased with larger SNR (smaller than 1 s for most SNR levels, and smaller than 0.5 s for SNR $>-4$ dB ). For the long duration HRF3, errors in TTP1 with HbO and HbR were overall larger (below 2 s at $-\,14$ dB and smaller than 1 s for all SNR $>\,-$10 dB), suggesting a less accurate estimation of the delay of the peak. However, it is worth noticing that most of these errors in TTP1 estimation were below 1 s and therefore quite reasonable when compared to fMRI temporal resolution.

Errors on the estimation of the undershoot peak (TTP2) are reported for all HRF models in Fig. 7 C for HbO and D for HbR. For all HRF models, errors in the estimation of TTP2 were overall larger than errors in the estimation of TTP1, and results presented more variability. Errors in the estimation of TTP2 decreased when increasing the SNR level. For HRF1, errors were below 4 s at − 14 dB and down to 2 s at 10 dB suggesting that robust estimation of the undershoot can only be obtained at high SNR levels. For HRF models 2 and 4, errors were smaller than 1 s for most SNR levels.

**Figure 8:**
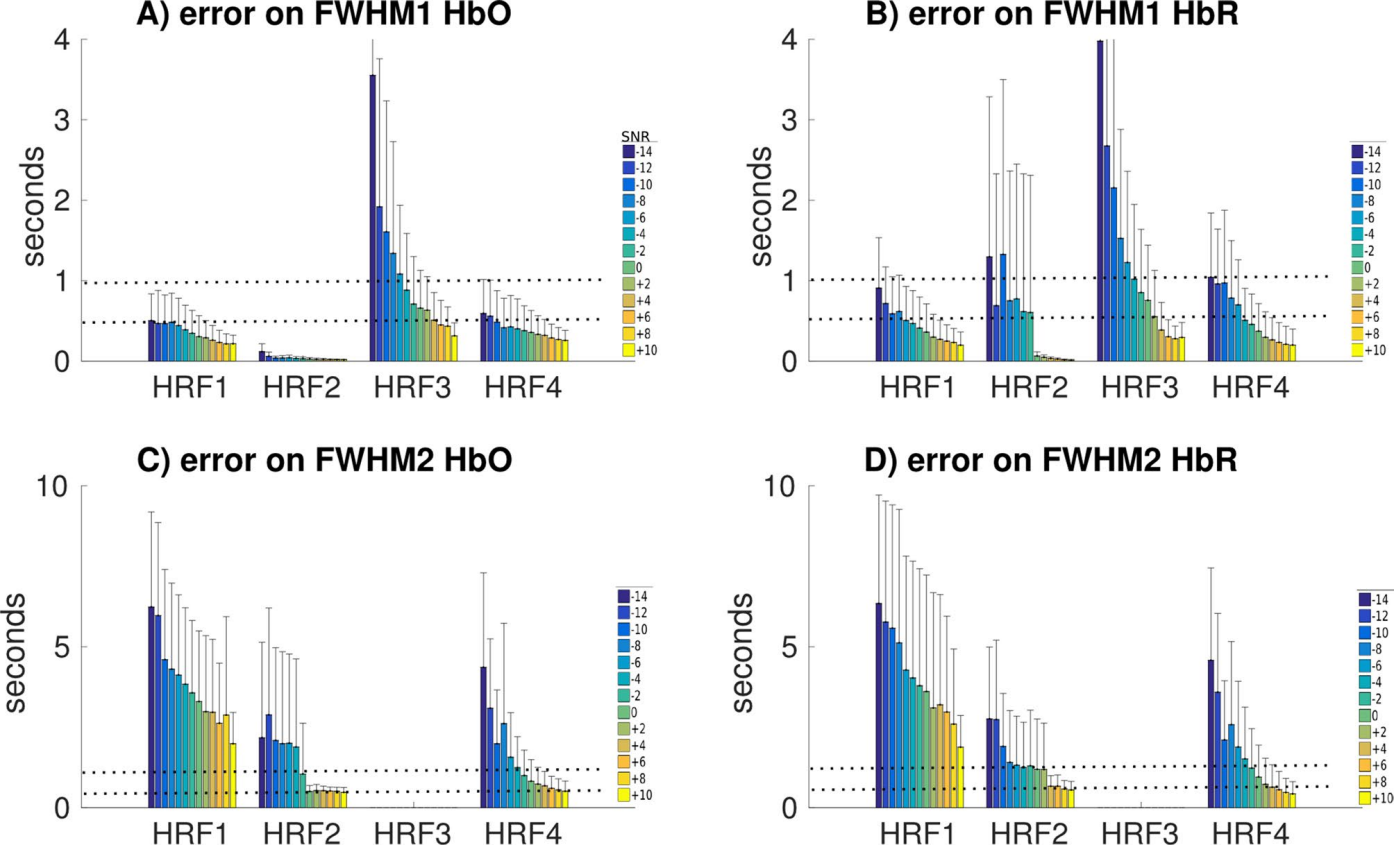
Distributions of the errors in the estimation of FWHM as a function of the SNR level for all HRF models. Note that FWHM2 for HRF3 was not estimated as no undershoot were simulated.

Error in the estimation of the durations of the main peak (FWHM1) and undershoot (FWHM2), are reported for all HRF models in Fig. 8. Errors in FWHM1 were for most SNR levels below 1 s for HRFs 1,2 and 4 and below 1 s for long duration HRF3 for SNR levels $>\,-2$ dB. We observed similar errors in FWHM2 for HRF 2 (short response) and 4 (large undershoot). However, for the canonical HRF1, errors in FWHM2 were larger than errors in FWHM1 (less than 6 s at − 14 dB, down to 2 s at 10 dB), more likely because the undershoot was simulated at a lower amplitude ratio when compared to HRF2 and HRF4 models (see Fig. 2).

### HRF deconvolution applied on DOT reconstruction of real fNIRS data

**Figure 9:**
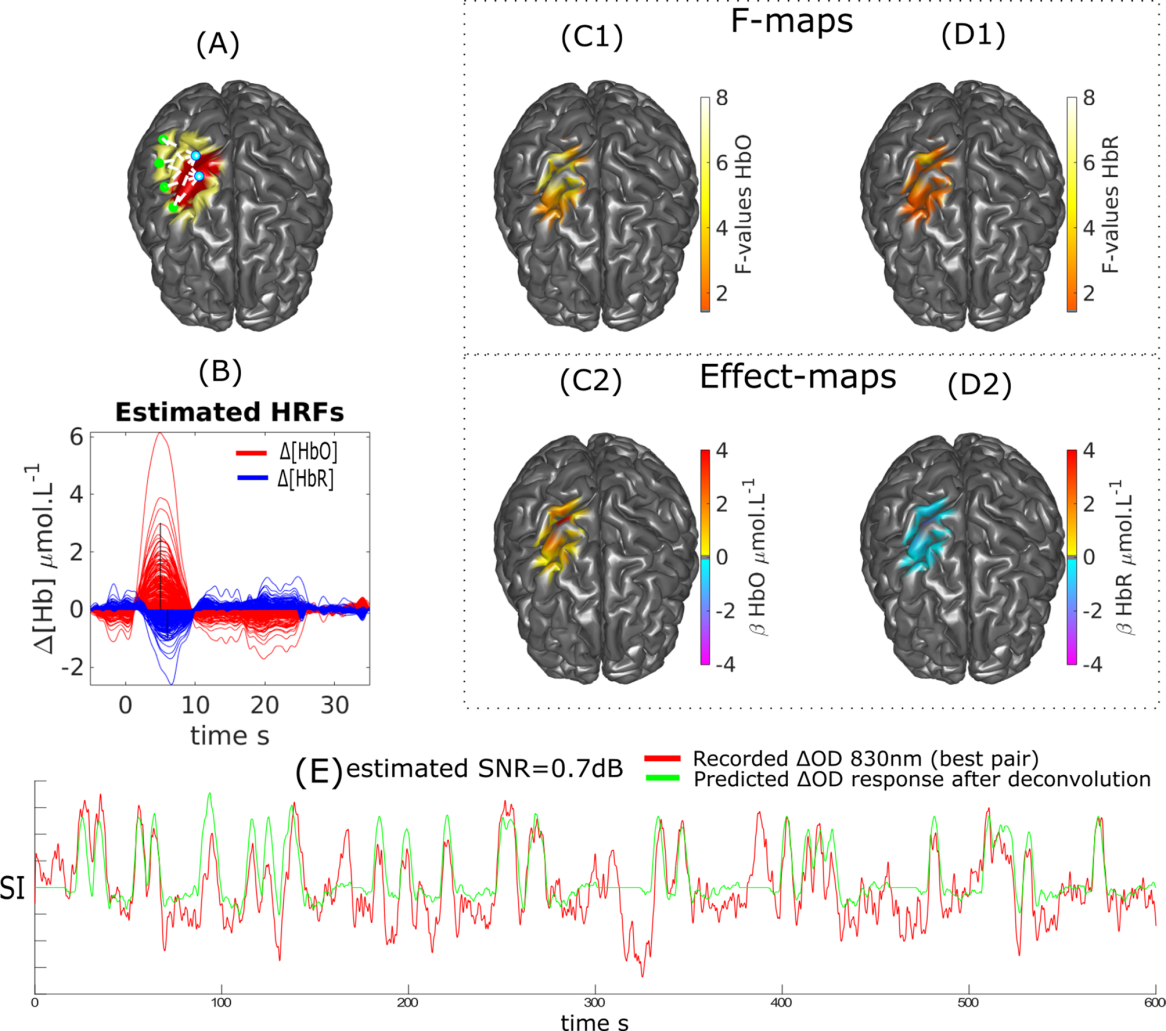
DOT reconstruction and HRF deconvolution for a finger opposition task on Subject 1. (**A**) Montage, ROI (red) and FOV (yellow). (**B**) Estimated HRF time courses. (**C1**, **C2**) $\Delta [HbO]$: Thresholded F-map ($\alpha =0.05$, Bonferronni corrected) and associated effect size map estimated at $TTP1=5$ s. (**D1**, **D2**) $\Delta [HbR]$: Thresholded F-map ($\alpha =0.05$, Bonferronni corrected) and associated effect size map estimated at $TTP1=6$ s. (**E**) Most sensitive $\Delta OD^{830}$ pair over the target ROI and corresponding predicted response after deconvolution (at the sensor level) with the binary stimuli vector.

**Figure 10:**
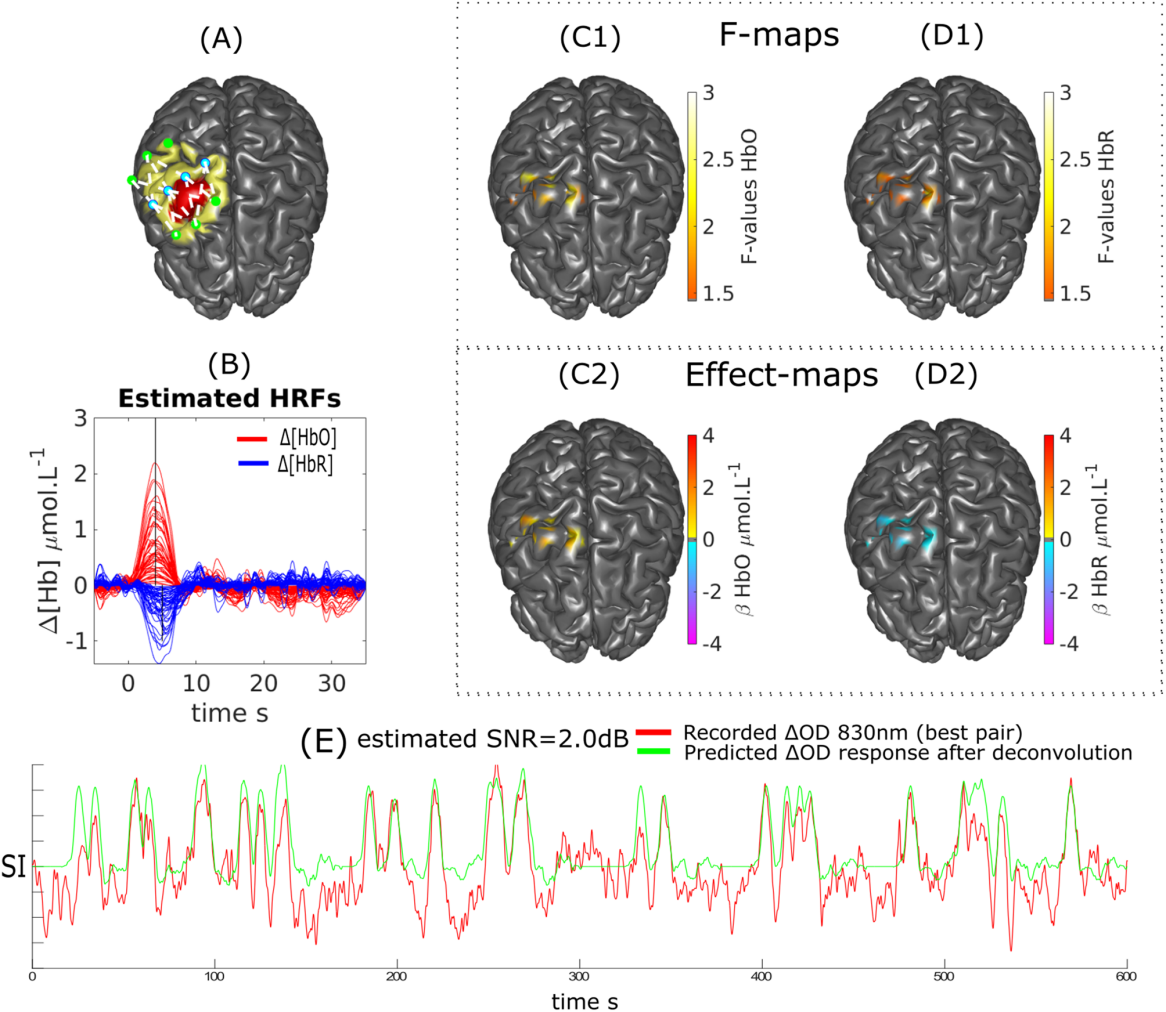
DOT reconstruction and HRF deconvolution for a finger opposition task on Subject 2. (**A**) Montage, ROI (red) and FOV (yellow). (**B**) Estimated HRF time courses. (**C1**, **C2**) $\Delta [HbO]$: Thresholded F-map ($\alpha =0.05$, Bonferronni corrected) and associated effect size map estimated at $TTP1=5$ s. (**D1**, **D2**) $\Delta [HbR]$: Thresholded F-map ($\alpha =0.05$, Bonferronni corrected) and associated effect size map estimated at $TTP1=6$ s. (**E**) Most sensitive $\Delta OD^{830}$ pair over the target ROI and corresponding predicted response after deconvolution (at the sensor level) with the binary stimuli vector.

Finger opposition results obtained on subjects 1 and 2 are presented in Figs. 9 and 10 respectively. The SNR of the most sensitive pairs to the target brain region were respectively estimated at 0.7 dB and 2 dB for these subjects. Responses were identified in the *ROI* targeted by our optimal montage strategy (i.e., hand knob area). Quantitatively, the main peaks of both HbO and HbR responses obtained after deconvolution were very close to the ones of a canonical HRF model, peaking around 5 s for HbO and 6 s for HbR. We observed a small undershoot estimated for subject 1 but none for subject 2.

**Figure 11:**
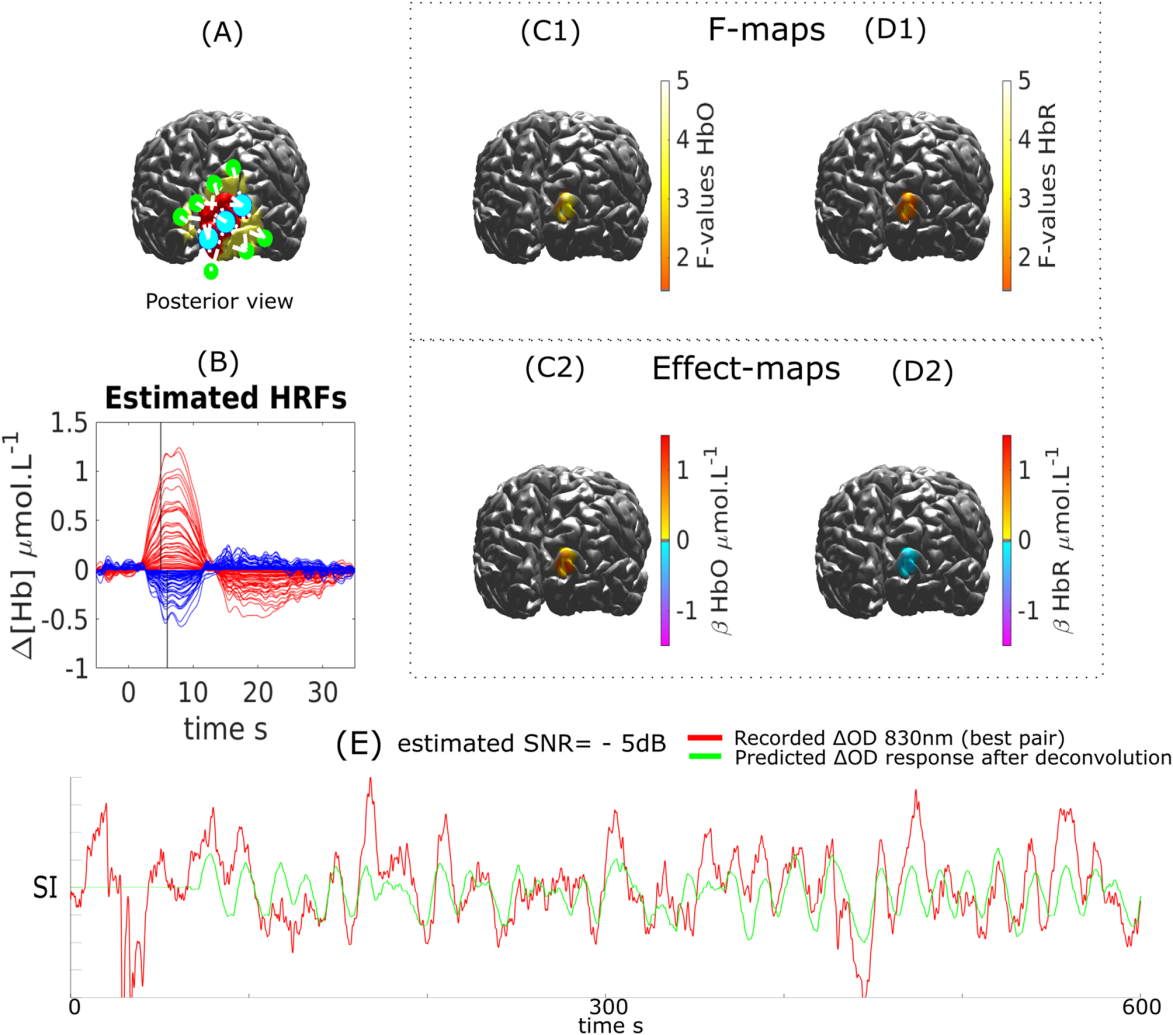
DOT reconstruction and HRF deconvolution for a visual task on Subject 3. (**A**) Montage, ROI (red) and FOV (yellow). (**B**) Estimated HRF time courses. (**C1**, **C2**) $\Delta [HbO]$: Thresholded F-map ($\alpha =0.05$, Bonferronni corrected) and associated effect size map estimated at $TTP1=5$ s. (**D1**, **D2**) $\Delta [HbR]$: Thresholded F-map ($\alpha =0.05$, Bonferronni corrected) and associated effect size map estimated at $TTP1=6$ s. (**E**) Most sensitive $\Delta OD^{830}$ pair over the target ROI and corresponding predicted response after deconvolution (at the sensor level) with the binary stimuli vector.

Visual task experiment on subject 3 is presented in Fig. 11. The SNR of the most sensitive pair was estimated at − 5 dB for this subject. Responses were identified in the *ROI* targeted by our optimal montage strategy (i.e., primary visual cortex). Quantitatively, the main peaks of both HbO and HbR responses obtained after deconvolution were very peaking around 6 s for both HbO and HbR.

**Figure 12:**
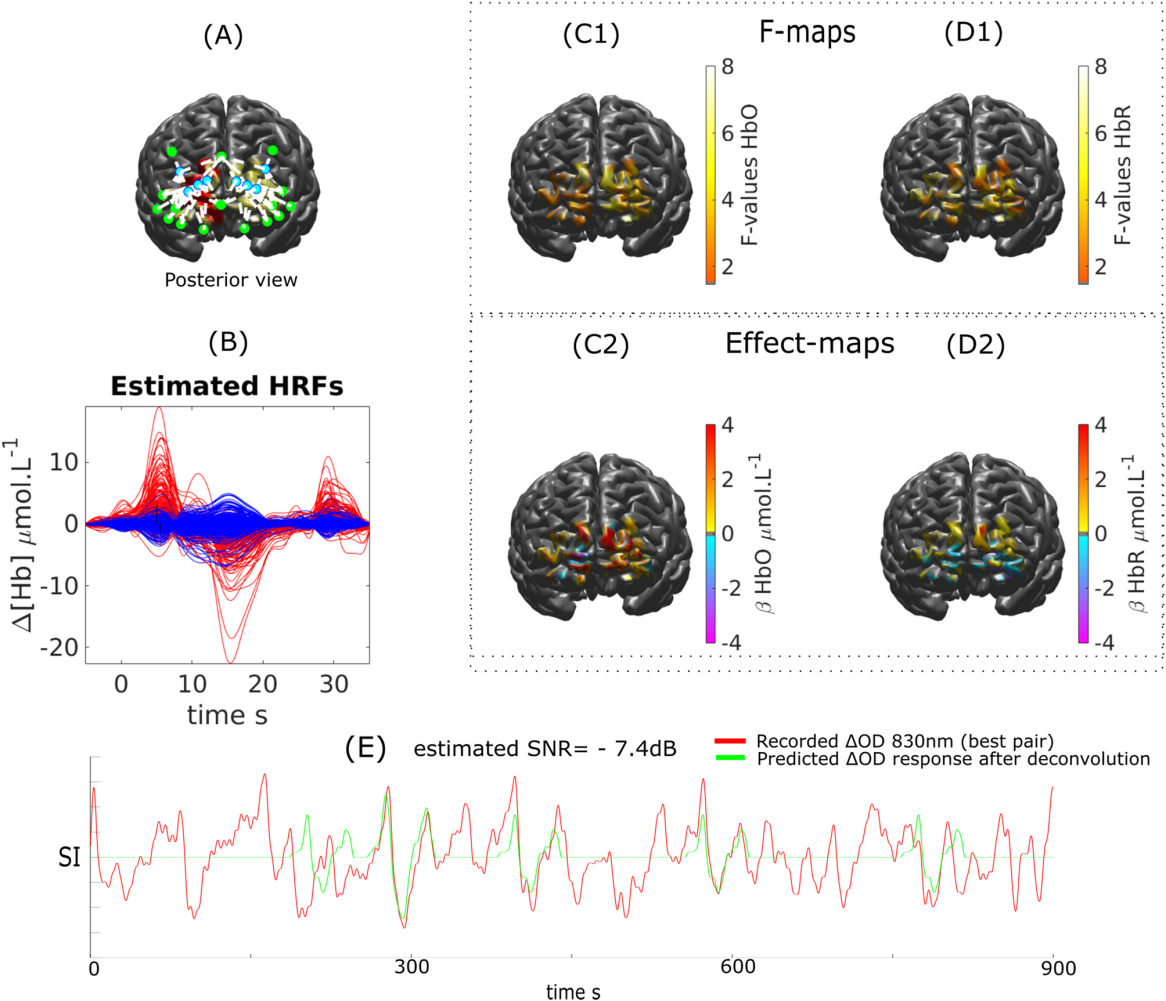
EEG/fNIRS investigation of a patients with right frontal epilepsy. DOT reconstruction and HRF deconvolution for HbO and HbR response following bursts of rapid activity. (**A**) Montage, ROI (red) and FOV (yellow). (**B**) Estimated HRF time courses. (**C1**, **C2**) $\Delta [HbO]$: Thresholded F-map ($\alpha =0.05$, Bonferronni corrected) and associated effect size map estimated at $TTP1=5$ s. D1,D2) $\Delta [HbR]$: Thresholded F-map ($\alpha =0.05$, Bonferronni corrected) and associated effect size map estimated at $TTP1=6$ s. (**E**) Most sensitive $\Delta OD^{830}$ pair over the target ROI and corresponding predicted response after deconvolution (at the sensor level) with the binary stimuli vector representing transient epileptic bursts.

fNIRS analysis of transient epileptic discharges (i.e., bursts of rapid activity) is presented in Fig. 12. We found significant HRFs for HbO and HbR to burst of rhythmic fast activity. In agreement with our findings reported in Pellegrino et al.^16^, we observed bilateral frontal significant hemodynamic responses. We report the global response, but individual responses measured in each hemisphere were not significantly different. The underlying mechanisms explaining the occurrence of those bilateral fNIRS responses to unilateral epileptic discharges, also reported in EEG/fMRI studies, are complex and not clearly understood. For HbO, the main peak of the reconstructed HRF (peak around 5–6 s and a duration of 8 s) was close to the canonical form with a very prominent undershoot peaking at 15 s. The small increase in HbO occurring at 30 s might be caused by some physiological contaminations. The SNR, computed retrospectively, was found to be at − 7.4 dB for the best pair. For HbR, we found a decrease in the inferior part of the FOV (peak around 5–6 s and a duration of 5 s). On the superior part of the FOV, we found a reverse pattern consisting in an increase HbR followed by a negative undershoot, which could suggest surround inhibition.

## Discussion

The purpose of this study was to evaluate robustness of HRF deconvolution after reconstruction of $\Delta [HbO]$ and $\Delta [HbR]$ along the cortical surface from fNIRS recordings. While deconvolution has been studied exclusively in the sensor space (i.e., among source-detector channels)^40,41^, it is the first time such a validation is performed after reconstruction along the cortical surface. One of the most important result is that deconvolution after inverse modelling using an AR(1)-MLE estimator seems to be robust, especially at realistic SNR levels (i.e., between − 5 and 5 dB for the most sensitive channel over the target ROI).

Through this study we used personalized optimal montages aiming at maximizing the sensitivity to a target brain region as they offer excellent properties for local reconstructions and provide overall better SNR in the source-detector space than non optimized sparse arrangements of optodes^15–18^. Our HRF deconvolution results should remain valid or even improve, when considering spatially extended ultra high density montage^6,13,14^ as these montages have even better properties for reconstructions.

We validated the proposed methodology in a simulation environment with scalp evoked *OD* changes obtained using cortical activation models. We considered 4 different shapes of HRF with (different peak amplitudes, durations, and delays) to investigate the ability of deconvolution techniques to recover a large spectrum of HRF shapes. We also varied the SNR of scalp measured *OD* changes applying a global scaling factor to real physiological and instrumental noise signals acquired at rest^46^. Here we are reporting the results obtained using simulations involving a rapid event related paradigm (30 trials, ITI: 2–60 s). We obtained similar performances when considering another event-related design or block designs for simulations (results not shown). This is in agreement with Aarabi et al.^40^ who demonstrated using a vast array of stimulus paradigms that the timing of events had negligible effects effect on the performance of deconvolution.

Overall, we obtained quite accurate reconstructions for HRFs for both HbO and HbR signals over a large range of SNR levels, whereas estimating HRF amplitude, delays and durations for HbR required larger SNR to reach similar accuracy than for HBO. Our results are overall suggesting that the HRF main peak can be measured with good accuracy under 1 s for long duration HRF models and excellent accuracy below 0.25 s for canonical, short and large undershoot HRF models. Similarly, we found that the main peak durations (i.e. FWHM) can be measured with good accuracy below 1 s for canonical, short and large undershoot HRF models. Our results are also suggesting that when the amplitude of the undershoot is large, delay and duration of the HRF can be measured with good accuracy below 1 s over a large range of SNR levels.

Long duration HRF models were more difficult to estimate accurately due to the intrinsic smoother variations of the response which may overlap easily with systemic physiological fluctuations. Aarabi et al.^40^, for instance, investigated the effect of confounding variables on HRF estimation using deconvolution at the sensor level in a rat model and demonstrated that periodic low-frequency systemic hemodynamic fluctuations as well as phase-locked noise can markedly obscure hemodynamic evoked responses in rapid event-related designs.

We demonstrated our ability to reconstruct event related responses to a finger opposition task or a flashing checkerboard using deconvolution after local reconstruction along the cortical surface. While we were able to reconstruct the main peak for HbO and HbR with sufficient confidence, the reconstruction of the undershoot was less accurate in agreement with our simulation results. We estimated that the SNR obtained on these real measurements was ranging from − 5 to 2 dB (Fig. 9, 10 and 11). When analysing fNIRS response to transient bursts of rhythmic activity in a patient with right frontal epilepsy, we found a significant HRF response corresponding to a large increase in $\Delta [HbO]$ followed by a large undershoot. Results for $\Delta [HbR]$ were more difficult to interpret, probably due to the lower SNR level reported (− 7.4 dB). Therefore improving the SNR and limiting the influence of systemic physiological fluctuations should be considered to allow more robust HRF estimations. While optimal montage has been proposed as a technique to increase the SNR^18^, improved SNR could be achieved through additional filtering techniques including PCA filtering (not considered here as the risk to remove cortical hemodynamics distributed over all channels of our local montage being too large) and superficial signal correction using short separation (< 5 mm) source-detector measurements^67–70^.

The main advantage of the proposed deconvolution method is to gain insights about neurovascular coupling mechanisms considering the possible underlying variability of the hemodynamic response. While our main objective was first to propose a detailed quantitative evaluation of the method. A detailed analysis of HRF variability at the intra- and inter-subject level is of great interest. We hope to carefully address these important questions in our future investigations on larger populations of patients and additional tasks.

Incorrect assumptions on the unexplained errors of the deconvolution model applied to fNIRS data containing serial-correlated noise and motion-related artefacts may also have led to inflated F test statistics, false positives activations around the ROI and incorrect estimation of the HRF. However, the lack of significant reconstructions below − 20 dB, which may be considered as a null condition, and our additional validation study using partial F test statistic (see supplementary material 1) suggested that our AR(1)-MLE with Bonferroni correction was appropriate for controlling type I errors after reconstruction. This result is indeed important since the AR(1)-MLE estimator is a computationally low cost estimator. Our results are in agreement with Huppert^71^ who suggested that AR(1) models should be sufficient when signals are almost free of motion artefacts, whereas higher order AR models should be considered in presence of motion. In our case, our recordings were almost free from any motion artefacts because we used collodion to maintain optode contacts on the skin. Investigation of different strategies to handle atypical error structures including higher AR orders^40,41,71,72^, precoloring^73^ or whitening techniques in the wavelet domain^24,44^ was out of the scope of this study but could be considered in the future. Additionally, future studies could include less conservative approaches to handle the multiple comparison problem such as the random field theory^24,28^ or the false discovery rate approach^74^.

Comparison of the FIR basis set with other basis sets or statistical formulation of the multiple linear regression problem within a Bayesian formalism^44,75,76^, allowing for instance the adjustment of the HRF smoothness level, was out of the scope of present study. In theory, other HRF frameworks with still a high degree of freedom (such as the gamma basis set) should result in similar performances after linear regression along the cortical surface as suggested in fNIRS at the sensor level^41^. However, low degree-of-freedom models, such as canonical HRF and its derivatives should be preferred only if we can tolerate a degree of mismatch between the underlying HRF shape and the model assumptions^41^.

Quantitatively, the true amplitude of the simulated activity was systematically misestimated (e.g., overestimated in the center and underestimated away). Indeed, it is generally accepted that the L2-minimum-norm model is tends to favour smeared solutions around locations where the measurements are the most sensitive, especially for EEG/MEG source localization^53^. Errors are still within values reported in Boas et al.^11^, Tian and Liu^27^ when using DOT on regular high density montages and within values reported by Machado et al.^18^ when applying inverse modelling with optimal montages. Better accuracy could be expected from more advanced inverse operators, such as the maximum entropy on the mean method^77^.

## Conclusion

In the present study, we investigated the accuracy HRF deconvolution when applied to reconstruction along the cortical surface obtained from personalized optimal montages that can be qualified as a local quantification tool with improved optical coupling and minimal movement sensitivity. While deconvolution is generally considered challenging, we demonstrated that HRF deconvolution, even using a straightforward computationally low cost AR(1)-ML estimator and F based inference, was able to accurately recover a large variability of HRFs along the cortical surface allowing to exploit the richness of the high temporal resolution offered by fNIRS. Our results indicate that particular attention should be placed upon maximizing SNR when designing fNIRS studies especially when the objective is to estimate low amplitude transients such as the undershoot.

## Supplementary Information

Supplementary Information.
