# Supplementary material for: Deconvolution of hemodynamic responses along the cortical surface using personalized functional near infrared spectroscopy
Source: Sci Rep. 2021 Mar 16;11:5964. doi: 10.1038/s41598-021-85386-0 (PMC7966407; doi:10.1038/s41598-021-85386-0)
Supplement: Supplementary file 1 — Supplementary Information. [file 41598_2021_85386_MOESM1_ESM.pdf]

Deconvolution of hemodynamic responses along the cortical surface using personalized functional near infrared spectroscopy

A Machado,[a] Z Cai,[a,b], T Vincent,[a,b] G Pellegrino,[c] J-M Lina,[d,e,f] E Kobayashi,[c], C Grova [a,b,c,e]

a Multimodal Functional Imaging Laboratory, Biomedical Engineering Department, McGill University, Canada
b Department of Physics and PERFORM center, Concordia University, Montreal, Canada
c Montreal Neurological Institute, Department of Neurology and Neurosurgery, McGill University, Canada
d École de technologie supérieure de l'Université du Québec, Canada
e Centre de Recherches en Mathématiques, Montréal, Québec, Canada
f Centre d'Etudes Avancées en Médecine Du Sommeil, Centre de Recherche de l'Hopital Sacré-Coeur De Montréal, Montréal, Québec, Canada
Address all correspondence to Dr Alexis Machado 3801 Rue University 751, Montréal, QC H3A2B4
alexis.machado@mail.mcgill.ca

March 4, 2021

Supplementary material 01: Specificity of the AR(1)-Maximum likelihood estimator using partial F statistic

In our study, HRF deconvolution was obtained using an AR(1)-Maximum Likelihood Estimator and inference conducted using a partial F statistic. It is debatable whether an AR(1) structure should be consider to model the unexplained errors of the deconvolution model (see eq.2.10) when applied to real fNIRS data.[1,2,3]. This is especially important as errors in modeling the unexplained errors of the model could result in inflated detection statistics and lack of specificity. Therefore, we decided to carefully investigate the empirical false positive rate of our estimator, building an empirical distribution of the null distribution of the partial F statistic. To do so, we generated surrogate $\Delta[HbO]$ or $\Delta[HbR]$ time series from resting state data using bootstrap resampling in the time-frequency domain in order to preserve data structure[4,5].

Generation of the surrogate dataset

We carefully selected in our database forty independent $\Delta[HbO]$ and $\Delta[HbR]$ channels (10 min at 5Hz) acquired at rest from different subjects. Source-detector separation for these channels was constrained between 30 and 35 mm. Amplitude and spectral content (heart beat, respiration and Mayer wave components) of the corresponding signals were carefully checked in order to ensure we selected good quality fNIRS. No movement artifacts were present in the data. From these 40 independent baselines, we applied bootstrap resampling in the wavelet domain[5,6] to generate surrogate datasets exhibiting the same statistical properties as the original resting state data. Indeed, the wavelet decomposition offers some intrinsic whitening properties[4] which are required for bootstrap procedures. Each selected baseline was first decomposed using a discrete wavelet transform algorithm[7] with Daubechies wavelets (4 vanishing moments). The discrete wavelet decomposition provides sets of uncorrelated

coefficients over the different scales. Symmetric boundary value replication was used to minimize border distortions. For each scale, ordinary bootstrap resampling (random sampling with replacement) was then applied to all detail coefficients. The approximation coefficients, which represents the very slow fluctuations of the signal in the wavelet decomposition (~ 0.007 Hz) were left unchanged. Finally, the inverse discrete wavelet transform was obtain to reconstruct surrogates time series. The whole resampling procedure was repeated 250 times for each of the 40 baselines (see Fig.1A), resulting in a set of $10,000$ surrogates of $\Delta[HbO]$ or $\Delta[HbR]$ signals following the H_0 distribution (data acquired at rest). Note that the same resampling were applied for HbO and HbR thus preserving the relationship between these two chromophores. As presented in Fig.1B, we applied spectral analysis to verify that our proposed resampling strategy preserved accurately the autocorrelation structure of the data (c.f. 1/f power spectrum density), even if few sharp spectral features as the cardiac peak in the $\Delta[HbO]$ signal could be dampened.

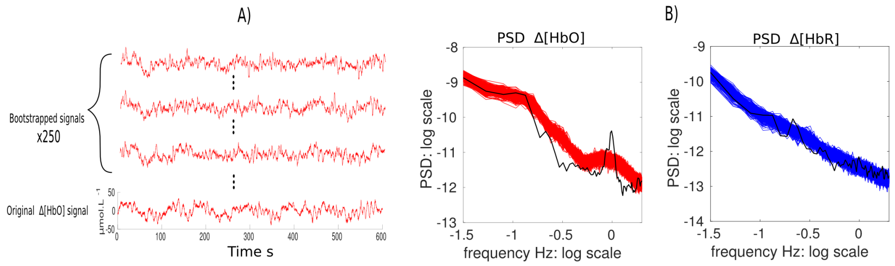

Figure 1: A) Illustration of bootstrap resampling in the time frequency domain for one $\Delta[HbO]$ time series. B) right: Power spectrum density of a selected $\Delta[HbO]$ time series (black line) and of the corresponding 250 resampled time series (red lines); left: Same representation for $\Delta[HbR]$.

Validation

For each signal from the surrogate dataset assumed to be distributed according to the null distribution H_0, we estimated $\hat{\boldsymbol{\beta}}_h$ and the corresponding partial F-statistics through deconvolution analysis using first an AR(0)-MLE (i.e., $\mathbf{\Omega} = \mathbf{I}$) then an AR(1)-MLE as decribed in the paper. The unknown HRF was modelled

by a FIR set between $[-5s\ 35s]$ sampled at $5Hz$ resulting in a set of $K = 201$ basis. An additional basis set of $M = 12$ cosine functions (up to 0.01 Hz) was also included in the design matrix to model the drifts. For evaluation of the specificity of our estimators, we inferred the presence of either a block paradigm or an event-related paradigm (2 example of each) from the surrogate resting state data. The empirical distributions of F values obtained using the 10,000 surrogate time series were compared with the theoretical Fisher's F probability density function under H_0 (cf. Eq.2.15). Considering the theoretical threshold $F_{th} = 1.18$ for H_0 rejection at a significance level of 5%, we estimated the empirical false positive rate, by counting the number of occurrence of significant results ($F > F_{th}$) over all surrogates.

Results

When performing the AR(0)-MLE assuming no temporal autocorrelation between residuals for our rapid event-related paradigm, we found that the empirical distribution of F-values diverged drastically from its theoretical probability density function (cf. Fig.2). The empirical false positive rate was estimated to be 32.5% for HbO and 38.2% for HbR. Similar rates were found for other event related and block related paradigms (not shown). On the other hand, when applying AR(1)-MLE on these bootstrap resampled baseline data, we found the empirical distribution of F-values to be in excellent agreement with the theoretical probability density function. For both HbO and HbR signals and for all paradigms, the empirical false positive rate was estimated to be $7.2\% \pm 0.8$, therefore very close to its expected value of 5%.

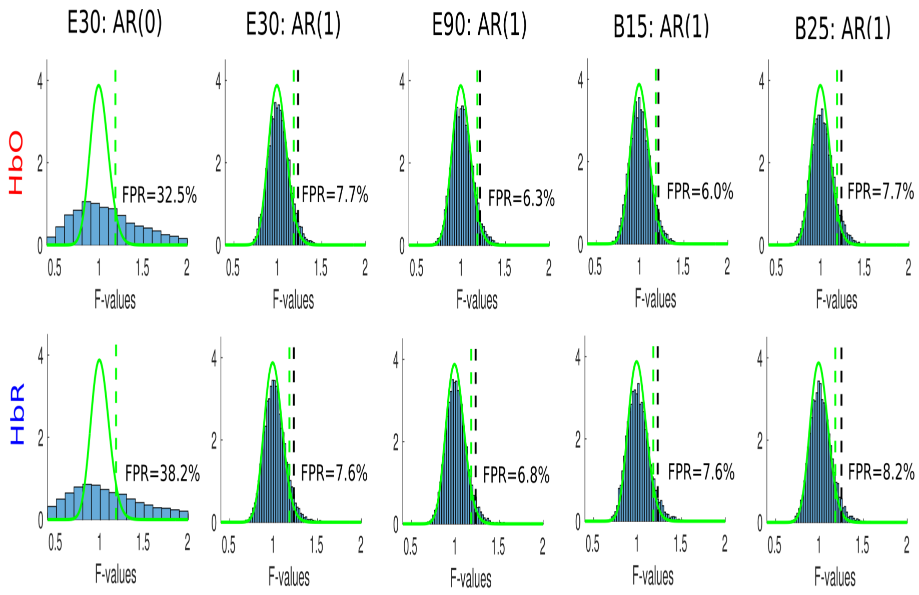

Figure 2: Distributions of F values obtained by applying either the AR(0)-MLE or AR(1)-MLE on 10,000 surrogates data following H_0 and considering different paradigms. E30: Event related design 1 (30 trials, interstimulus interval: 2 to 60s); E90: Event related design 2 (90 trials, interstimulus interval: 2 to 23s); B15: Block design 1 (10 trials of 15s duration , interstimulus interval : 30s); B25: Block design 2 (10 trials of 25s, interstimulus interval: 50s). Histograms were normalized as probability density functions. The theoretical Fisher's F ($df1 = 201, df2 = 2787$) probability density function is presented in green. Green and black vertical dashed lines represent respectively the theoretical ($F_{th} = 1.18$) and empirical statistical thresholds for H_0 rejection at 5% of significance. The empirical False Positive Rate (FPR) is shown in each graph.

References

[1] T. Fekete, D. Rubin, J. M. Carlson, and L. R. Mujica-Parodi. The NIRS Analysis Package: Noise Reduction and Statistical Inference. *PLOS ONE*, 6(9):e24322, September 2011. doi: 10.1371/journal.pone.0024322.

[2] J. W. Barker, A. Aarabi, and T. J. Huppert. Autoregressive model based algorithm for correcting motion and serially correlated errors in fNIRS. *Biomedical Optics Express*, 4(8):1366–1379, July 2013. doi: 10.1364/BOE.4.001366.

[3] T. J. Huppert. Commentary on the statistical properties of noise and its implication on general linear models in functional near-infrared spectroscopy. *Neurophotonics*, 3(1):010401–010401, 2016. doi: 10.1117/1.NPh.3.1.010401.

[4] P. Flandrin. Wavelet analysis and synthesis of fractional Brownian motion. *IEEE transactions on information theory*, 38:910–917, 1992.

[5] E. Bullmore, J. Fadili, M. Breakspear, R. Salvador, J. Suckling, and M. Brammer. Wavelets and statistical analysis of functional magnetic resonance images of the human brain. *Statistical Methods in Medical Research*, 12(5):375–399, October 2003. doi: 10.1191/0962280203sm339ra.

[6] O. Friman and C.-F. Westin. Resampling fMRI time series. *NeuroImage*, 25 (3):859–867, April 2005. doi: 10.1016/j.neuroimage.2004.11.046.

[7] S. G. Mallat. A theory for multiresolution signal decomposition: the wavelet representation. *IEEE transactions on pattern analysis and machine intelligence*, 11(7):674–693, 1989.

List of Figures

1 A) Illustration of bootstrap resampling in the time frequency domain for one $\Delta[HbO]$ time series. B) right: Power spectrum density of a selected $\Delta[HbO]$ time series (black line) and of the corresponding 250 resampled time series (red lines); left: Same representation for $\Delta[HbR]$. 3

2 Distributions of F values obtained by applying either the AR(0)-MLE or AR(1)-MLE on 10,000 surrogates data following H_0 and considering different paradigms. E30: Event related design 1 (30 trials, interstimulus interval: 2 to 60s); E90: Event related design 2 (90 trials, interstimulus interval: 2 to 23s); B15: Block design 1 (10 trials of 15s duration , interstimulus interval : 30s); B25: Block design 2 (10 trials of 25s, interstimulus interval: 50s). Histograms were normalized as probability density functions. The theoretical Fisher's F ($df1 = 201, df2 = 2787$) probability density function is presented in green. Green and black vertical dashed lines represent respectively the theoretical ($F_{th} = 1.18$) and empirical statistical thresholds for H_0 rejection at 5% of significance. The empirical False Positive Rate (FPR) is shown in each graph. 5

List of Tables
